# Vestibulo-Oral inclination of maxillary and mandibular canines and bicuspids - a CBCT investigation

**DOI:** 10.1186/s13005-016-0119-8

**Published:** 2016-07-07

**Authors:** Jan Hourfar, Dirk Bister, Jörg A. Lisson, Christine Goldbecher, Björn Ludwig

**Affiliations:** Department of Orthodontics, University of Heidelberg, Heidelberg, Germany; Department of Orthodontics, Guy’s and St Thomas’ NHS Foundation Trust and King’s College London Dental Institute, London, UK; Department of Orthodontics, University of Saarland, Homburg/Saar, Germany; Private Practice, Halle/Saale, Germany; Private Practice, Am Bahnhof 54, 56841 Traben-Trarbach, Germany

**Keywords:** Cone beam computed tomography, Tooth axis, Crown axis, Vestibulo-oral inclination, Torque

## Abstract

**Background:**

The aim of this retrospective study was to measure tooth and crowns axes of canines, first and second bicuspids of orthodontically untreated subjects with near normal occlusion to: 1. Define norms and reveal potential gender differences and 2. Discuss implications of the findings for orthodontics.

**Methods:**

The CBCT-datasets of 167 patients, 56 males (mean age 28.63 years ± 11.99 years) and 111 females (mean age 29.72 years ± 11.47 years) were used. Tooth- and crown axes were measured for right and left sides. Normal distribution was evaluated with the Kolmogorov-Smirnov-test. For gender comparison independent t-Tests and for comparison of right and left sides a paired *t*-Test were used for normally distributed data. For data not following normal distribution for gender comparison the Mann-Whitney-U-Test was used and for data comparing the two sides the Wilcoxon signed rank test was applied.

The level of statistical significance was set at *p* ≤ 0.05.

**Results:**

Measurement of tooth axes revealed buccal inclination for both genders with maximum values for maxillary and mandibular canines. Statistical significant differences were only found for maxillary canines (*P* = 0.025) and lower second bicuspids (*P* = 0.016) respectively. Values for crown axes revealed oral inclination for both genders with maximum values for maxillary first bicuspids and in the mandible for first and second bicuspids. No statistical significant differences were found between the genders apart from asymmetry for crown axes for the upper first bicuspids for males (*P* = 0.006) and females (*P* < 0.001).

**Conclusions:**

Our study reveals that irrespective of gender, oral inclination of the crowns of canines and premolars is the norm. The values of the most commonly used bracket prescriptions coincide with the average values found in our investigation. For esthetic reasons modifications of torque values can be considered.

## Background

Orthodontics aims at achieving good functional occlusion and dental aesthetics [1, 2]. A fundamental aspect to achieving good aesthetics and function is the predictable three-dimensional positioning of teeth. To accomplish this, contemporary orthodontics mostly relies on using pre-adjusted standard edgewise fixed appliances that are available with a range of different bracket prescriptions and slot sizes. By using these different prescriptions different torque values are applied.

Although not the sole determinant [3] of smile aesthetics, the vestibulo-oral inclination of the canines and bicuspids, particularly in the upper jaw [4], play an important role for the smile aesthetics. Approximately 90 % of people show either the maxillary first and second premolar when smiling [5] and the vestibulo-oral inclination of the maxillary canines and bicuspids can influence the width of the buccal corridor [4–6]. The buccal corridor is defined as the space between the facial surfaces of the posterior teeth and the corners of the lips when smiling [7]. Whether smaller or wider buccal corridors are preferable has been debated before [8] and there is some evidence suggesting gender differences [9].

Some authors recommend to give upper canines and bicuspids buccal crown torque to improve aesthetics [5]. However a number of commonly used bracket prescriptions apply negative torque to canines and bicuspids, causing oral inclination. A recent study by Xu et al., [4] however, investigating three dimensional digital models revealed a broad range of esthetic acceptability for vestibulo-oral inclinations of the maxillary canines and premolars. Vestibulo-oral inclination of crowns can be measured on study models or with Cone Beam Computed Tomography (CBCT) images. Only the latter are able to measure tooth axis and crown inclination with precision [10–12], because CBCT images show the roots as well as adjacent dentofacial structures undistorted in a 1:1 ratio [13]. Conventional two dimensional radiographs do not allow for precise measurements of bucco-lingual inclination of teeth [14].

The aim of this retrospective study was to measure vestibulo-oral inclination of roots and crowns of canines, first and second bicuspids in both jaws of orthodontically untreated subjects to:

1. Define norms and reveal possible gender differences and
2. Discuss implications for orthodontic treatment.

## Methods

Definition of abbreviations can be found in Table 1.

**Table 1:** Abbreviations

Abbreviation	Type	Definition
mrp	RPL	Median reference plane, aligned parallel to the dental arch of the split axial view.
ap	RP	apex. Most apical point of the root.
cej	RP	cemento-enamel-junction
i	RP	cusp tip
cf	RP	Central fossa. Deepest occlusal notch between cusps in bicuspids
FA	RP	FA-Point according to Andrews
α	AM	Tooth axis: Angle between long axis of the tooth (i-ap and cf-ap respectively) and midsagittal plane (msp).
β	AM	Crown axis: Angle between long axis of the tooth (i-ap and cf-ap respectively) and tangent through FA-point (constructed using parallel shift of the connective line between cej and i).

*RPL* reference plane, *RP* reference point, *AM* angular measurement (degrees)

### Patients and radiographic material

Anonymized, relevant CBCT images that had been taken between 2009 and 2012 were analyzed; the images were sourced from a practice that specializes in orthodontics and oral surgery.

Inclusion criteria were:

- Justification for the radiographs and written consent were available.
- The indications for imaging included: diagnosis of intraosseus and dental pathologies and both pre-operative risk assessment and surgical planning for various interventions, complying with the “as low as reasonably achievable” (ALARA) principle [15].

### CBCT-scans

The CBCT scans used in this study were all taken with the same equipment: Veraviewpocs 3D®, (J. Morita Corp., Osaka, Japan). Images were acquired with the following settings: 5 mA, 80 kV, pixel size: 0.125 mm × 0.125 mm; voxel size was 0.125 mm^3^. All CBCT images provided a slice thicknesses of 0.25 mm. Patients were positioned according to the manufacturer’s instructions.

### Patient selection and inclusion criteria

CBCT datasets of a total of 1007 patients were available. Only datasets with field of view (FOV) including complete dentition of both jaws without artifacts and only patients of Caucasian origin without history of previous orthodontic treatment were included. Further inclusion criteria included presence of fully erupted teeth that exhibited neither prosthetic restorations/fillings or dental caries. Only patients with near normal occlusion (NNO) were included. NNO was verified using available plaster models.

### Measurements on CBCT datasets

#### Software

All measurements were performed using DICOM imaging software (OsiriX®, Version 2.0.1, 64 Bit, Pixmeo, Bernex, Switzerland) for MacOS® (Apple Inc., Cupertino, Ca, USA). The software features 3 split windows for coronal, sagittal and axial view. After screening of the respective 3D-data sets, orthoradial adjustments to the x-, y- and z-plane level were made to enable reproducible three-dimensional measurements. The validity and accuracy of measurements performed with Osirix® software have been demonstrated by various previous studies [16–22].

#### Dental measurements

**Fig. 1:**
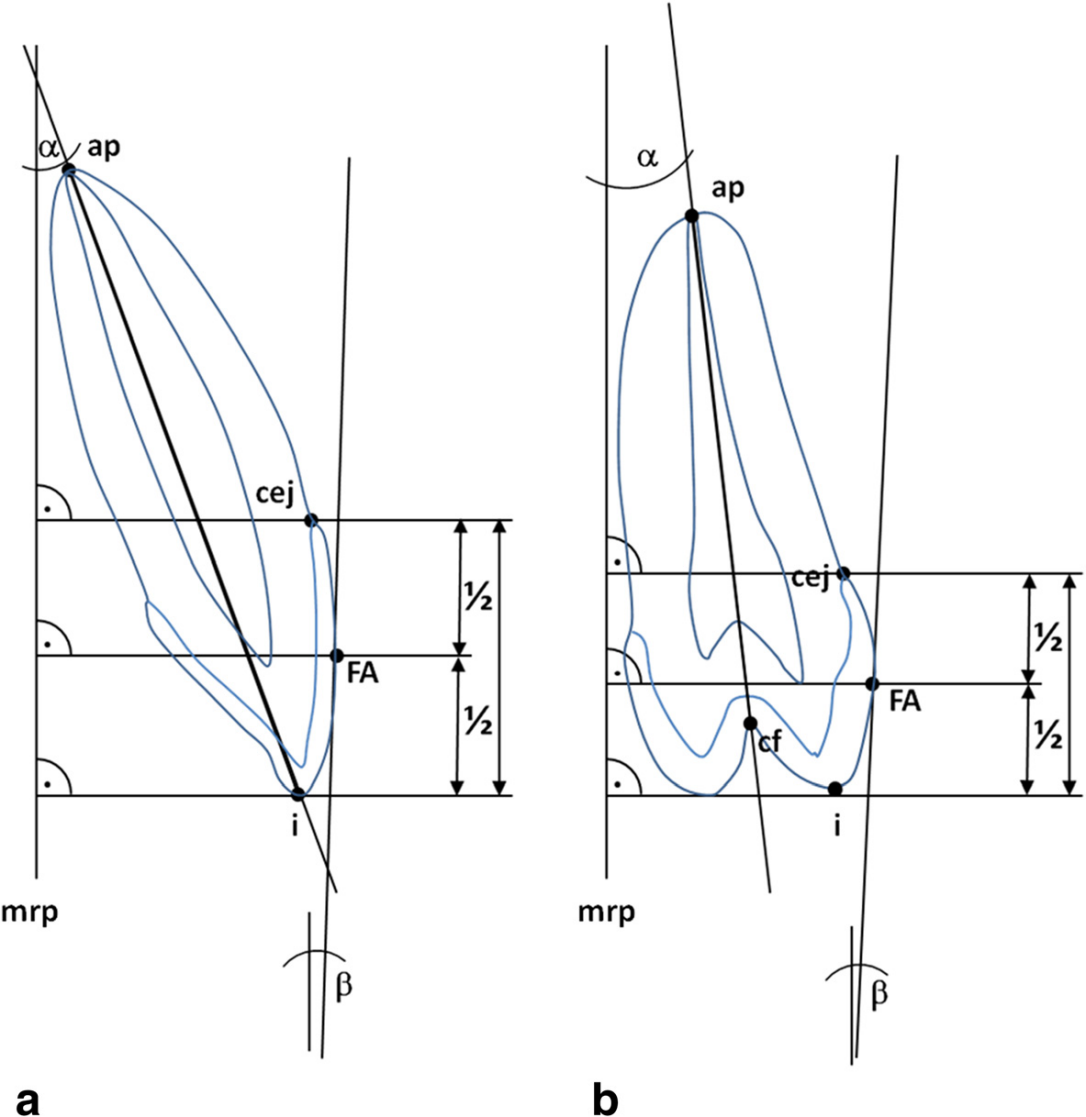
Measurement of crown and tooth axes. Measurements were undertaken using different reference points as defined in Table 1. Left upper canine (**a**) and left bicuspid (**b**) are shown here. Negative values (-) indicate oral inclination of crown or root whereas positive (+) values indicate vestibular inclination

The dental measurements consisted of 1. crown and 2. tooth axis. These two different measurements were performed using a median reference plane and a set of radiographic reference points and lines (Fig. 1, Table 1). The exact protocol for the measurement of crown axes and tooth axes are described in detail below. Tooth and crown axes were measured for canines, first bicuspids, and second bicuspids in both jaws. A section through each tooth measured it at its widest occlusal vestibulo-oral distance and was adjusted on the axial and sagittal split window respectively. All angular measurements were undertaken on the coronal split window of the imaging software using the built in angle measuring tool. Negative values indicate oral inclination of crown or root respectively whereas positive values indicate vestibular inclination of crown or root respectively. Mandibular and maxillary measurements were undertaken in the same way.

1. Crown axis
  The crown axis (i.e. the vestibulo-oral inclination of the crown) was defined and measured as the angle between the median reference plane (“mrp”) and the line through the FA-point according to Andrews [23]. As described by Smith et al. [24] a parallel shift of the connective line between the clearly defined reference points “cej” (cemento-enamel-junction) and “i” (cusp tip) for was used to construct the line through FA.
2. Tooth axis
  The tooth axis (i.e. the vestibulo-oral inclination of the root) was measured as the angle between the median reference plane (“mrp”) and a line passing through the cusp tip (“i”) of the canines (or “cf” of the bicuspids) and the apical reference point (“ap”). In case of root apex dilaceration, the middle third of the root was used as the apical reference point, similar to conventional cephalometry.

### Data collection and statistical analysis

All data were collated on an Excel® spreadsheet (Microsoft Corp., Redmond, Washington, USA). Statistical analyses were carried with SPSS® for Windows, version 22.0 (IBM Corp., Armonk, New York, USA). Normal distribution was evaluated with the Kolmogorov-Smirnov-test. For gender comparison independent t-Tests and for comparison of right and left sides a paired *t*-Test were used for normally distributed data. For data not following normal distribution for gender comparison the Mann-Whitney-*U*-Test was used and for data comparing the two sides the Wilcoxon signed rank test was applied. Descriptive statistics (Medians and Interquartile Ranges (IQR)) are presented for data not following normal distribution whereas descriptive statistics (Means and Standard Deviations (SD)) are presented for normally distributed data.

For intra-examiner reliability the same operator repeated all measurements for 50 randomly selected cases 3 months after the initial measurements and the coefficient of variation (COV) was calculated. The COV was mean 0.13 (range: 0.03–0.34) for males and mean 0.17 (range: 0.03–0.72) for females. No statistical difference (*P* = 0.554) was found between the COVs. The level of statistical significance was set at *p* ≤ 0.05.

## Results

### Sample demographics

A total of 167 patients, 56 males (mean age 28.63 years ± 11.99 years) and 111 females (mean age 29.72 years ± 11.47 years) fulfilled the criteria for inclusion. No statistically significant difference (*P* = 0.569) was found between age of both genders.

### CBCT-measurements

Descriptive values of the CBCT measurements and results of the statistical analysis are presented in Tables 2, 3, 4 and 5. Mean and median values for tooth axes presented buccal inclination for males and females with maximum values for maxillary and mandibular canines. Statistical significant gender differences were only found in maxillary canines (*P* = 0.025) and lower second bicuspids (*P* = 0.016) respectively (Tables 2 and 3). Conversely, mean and median values for crown axes revealed oral inclination for both genders with maximum values for maxillary first bicuspids and in the mandible for first and second bicuspids. No statistical significant differences were found between the genders. Interestingly there was statistically significant asymmetry for crown inclinations for the upper first bicuspids for males (*P* = 0.006) and females (*P* < 0.001) (Tables 4, 5).

**Table 2:** Tooth axes (in degrees) - *males*

Tooth	Mean, R	SD, R	Mean, L	SD, L	Avg (R, L)	SD, avg (R, L)	Mean diff (R-L)	*P* value (R vs L)	*P* value (M vs F)
U3	+12.62	6.96	+12.19	7.66	+12.40	7.28	0.43	0.668	0.025*
U4	+3.36	6.67	+3.99	6.17	+3.67	6.41	−0.64	0.636	0.237
U5	+4.33	7.14	+3.83	5.78	+4.09	6.48	0.50	0.374	0.190
L3	+17.41	7.44	+18.88	8.02	+18.15	7.73	−1.47	0.336	0.114
L4	(+5.24)	(7.79)	(+3.14)	(7.87)	(+3.40)	(7.84)	(2.10)	0.056	0.526
L5	(+6.20)	(7.23)	(+4.36)	(8.23)	(+5.58)	(8.24)	(1.84)	0.149	0.016*

*P ≤ 0.05; not normally distributed data in brackets

*R* right, *L* left, *Avg* average, *diff* difference, *M* male, *F* female, *U* upper, *L* lower; 3, canine; 4, first bicuspid; 5, second bicuspid

**Table 3:** Tooth axes (in degrees) - *females*

Tooth	Mean, R	SD, R	Mean, L	SD, L	Avg (R, L)	SD, avg (R, L)	Mean diff (R-L)	*P* value (R vs L)	*P* value (M vs F)
U3	+14.96	6.89	+14.47	8.19	+14.71	7.55	0.48	0.293	0.025*
U4	(+3.10)	(7.50)	(+2.58)	(7.12)	(+2.63)	(8.00)	(0.52)	0.387	0.237
U5	+2.61	7.48	+3.41	6.43	+2.99	6.99	−0.80	0.147	0.190
L3	+20.33	7.33	+19.32	8.77	+19.83	8.07	1.01	0.098	0.114
L4	+4.22	5.67	+3.56	5.47	+3.89	5.56	0.67	0.283	0.526
L5	+8.55	7.20	+7.16	6.44	+7.85	6.84	1.39	0.212	0.016*

*P ≤ 0.05; not normally distributed data in brackets

*R* right, *L* left, *Avg* average, *diff* difference, *M* male, *F* female, *U* upper, *L* lower; 3, canine; 4, first bicuspid; 5, second bicuspid

**Table 4:** Crown axes (in degrees) - *males*

Tooth	Mean, R	SD, R	Mean, L	SD, L	Avg (R,L)	SD, avg (R,L)	Mean diff (R-L)	*P* value (R vs L)	*P* value (M vs F)
U3	−6.23	6.01	−6.98	5.89	−6.61	5.92	0.74	0.426	0.358
U4	−8.66	7.40	−11.74	6.97	−10.12	7.32	3.08	0.006**	0.944
U5	−7.17	6.73	−7.80	5.95	−7.48	6.48	0.63	0.069	0.668
L3	(−0.37)	(5.22)	(−0.03)	(5.70)	(−0.36)	(5.79)	(−0.34)	0.981	0.345
L4	−11.35	8.25	−12.47	9.37	−13.08	8.77	1.12	0.540	0.743
L5	−13.53	8.50	−10.54	7.83	−12.98	8.23	−2.99	0.149	0.734

**P ≤ 0.01; not normally distributed data in brackets

*R* right, *L* left, *Avg* average, *diff* difference, *M* male, *F* female, *U* upper, *L* lower; 3, canine; 4, first bicuspid; 5, second bicuspid

**Table 5:** Crown axes (in degrees) - *females*

Tooth	Mean, R	SD, R	Mean, L	SD, L	Avg (R,L)	SD, avg (R,L)	Mean diff (R-L)	*P* value (R vs L)	*P* value (M vs F)
U3	−7.08	7.19	−6.76	7.02	−7.43	6.72	−0.32	0.075	0.358
U4	−8.48	6.24	−11.80	6.08	−10.06	6.36	3.32	<0.001***	0.944
U5	−7.79	7.48	−7.97	6.30	−7.91	6.83	0.18	0.898	0.668
L3	(−0.65)	(4.81)	(−1.55)	(8.15)	(−1.00)	(5.94)	(0.91)	0.104	0.345
L4	−11.67	7.06	−11.53	8.28	−12.69	7.69	−0.14	0.309	0.743
L5	−12.18	8.04	−12.54	7.94	−13.40	8.03	0.35	0.369	0.734

***P ≤ 0.001; not normally distributed data in brackets

*R* right, *L* left, *Avg* average, *diff* difference, *M* male, *F* female, *U* upper, *L* lower; 3, canine; 4, first bicuspid; 5, second bicuspid

## Discussion

The aim of our study was to define norms for vestibulo-oral inclination of teeth for an untreated Caucasian population, to investigate gender differences and to discuss possible implications of the findings for orthodontic treatment.

The results of our study referring the tooth axes were consistent with those of another CBCT study using similar methodology [13]; although neither crown axes nor gender differences were investigated by that group. The only difference were the lower second bicuspids, which showed vestibular inclination. Our study investigated only Caucasian patients whereas the sample assessed by Tong et al. [13] was comprised of 6 ethnicities: Hispanic, Black, White, Asian and Middle Eastern; Caucasian white patients constituted their smallest group and the differences between the ethnicities were not investigated.

In our study two comparisons between genders referring to tooth axes reached the level of statistical significance; these differences were likely to be spurious however. There was no difference between right and left that reached level of significance.

Crown axes did not exhibit statistically significant differences (*P* > 0.05) between male and female subjects and demonstrated oral inclination. It is interesting to note that a number of widely used prescriptions of commercially available bracket systems have negative torque values for canines and bicuspids: negative torque values (-7) can be found for maxillary canines (Andrews and MBT prescriptions) as well as for maxillary bicuspids (Roth, MBT, and Andrews prescriptions) [25]. Except for maxillary first bicuspids, the results of our study for crown axes of maxillary canines and second bicuspids resemble the torque values of the aforementioned prescriptions (Tables 2, 3). Interestingly our study showed asymmetry of the upper first premolar torque values between right and left hand sides for both males and females indicating asymmetry of approximately three degrees (−8.66 and −11.74 for males and −8.48 and −11.80 for females). This has to our knowledge not previously been described.

One factor contributing to an attractive or esthetic smile is the size of the buccal corridor [7] and numerous papers have been published on this [6, 9, 26–32] and the literature is inconclusive. In a systematic review, Janson et al. [33] pointed out that the influence of the buccal corridor on a smile was thought more important if digitally modified patient photographs were used for evaluation, rather than natural images; however a broader smile was preferred by most authors [5, 34]. Another study found smaller buccal corridors for male subjects and larger buccal corridors for female subjects aesthetically more pleasing [9], suggesting a gender difference.

Our study supports the notion that oral inclination of the maxillary bicuspids is the norm; approximately −7.5° for first and −10° for second bicuspids. An earlier investigation speculated that application of buccal crown torque to canines and posterior teeth might alleviate pronounced buccal corridors and enhance esthetics [5]. If application of buccal crown torque is desired, applying more positive values will subsequently move the roots of the teeth palatal potentially reducing the risk of developing vestibular bony dehiscence or recession [35].

A recent study found that orthodontists prefer ranges of 0° to −7° of vestibulo-oral inclination for the canines and −3**°** to −11° for the bicuspids esthetically pleasing. For laypersons the values were +3 to −10° for the canines and +5 to −11° of inclination for bicuspids [4]. Our investigation appears to confirm that orthodontists prefer naturally occurring inclinations of teeth, in contrast to the lay population.

Indiscriminate treatment of patients with a pre-adjusted standardized straight wire fixed appliances, using commercially available brackets and archwires is not consistent with individualized treatment. However torque prescriptions ‘programmed’ in bracket systems usually not fully expressed. This can be due to a variety of factors such as: inaccuracies of bracket positioning, differences in tooth morphology between individuals, because of torque loss (the ‘play’ between the archwire and slot) or the properties of the orthodontic materials themselves [36, 37]. Our study confirms that the torque values used in most commercially available bracket prescriptions are found in the untreated population.

The need for individualized treatment of the patient may be particularly interesting when considering extractions: One CBCT study demonstrated that non-extraction treatment increased the buccal crown torque of the upper bicuspids but that extraction treatment lead to lingual crown torque of upper canines [11].

Aesthetic considerations aside, the functional occlusion must not be neglected. Applying buccal crown torque to maxillary canines and bicuspids for esthetic reasons might interfere with functional occlusal contacts: canine guidance might be lost and for maxillary bicuspids the palatal cusps can interfere during lateral excursion and our study appears to suggest that orally inclined teeth are the norm for the untreated population. Although not part of this investigation we can speculate that a mutually balanced and protected occlusion may well be the norm. It has been recommended that post-treatment occlusion should be subjected to dynamic evaluation as well as the commonly used static assessment of the occlusion [38]; particularly with regard to desired postorthodontic treatment outcome: the majority of the referring dentists rank canine guidance as most important feature of the occlusion [39].

## Conclusions

- Our study revealed that irrespective of gender, oral inclination of canine and premolars crowns were the norm for the Caucasian white population investigated.
- The torque values of most commonly used bracket prescriptions coincide with the average values found in our investigation.
- There was an asymmetry in upper first premolar torque for both males and females that has not previously been reported.
